# Mechanistic and Kinetic Insights into the Acylation Reaction of Hepatitis C Virus NS3/NS4A Serine Protease with NS4B/5A Substrate

**DOI:** 10.3390/biom15111619

**Published:** 2025-11-18

**Authors:** José Ángel Martínez-González, Nuria Salazar-Sanchez, María Larriva-Hormigos, Rodrigo Martínez, Miguel González

**Affiliations:** 1 Departamento de Química y Bioquímica, Facultad de Farmacia, Universidad San Pablo-CEU, CEU Universities, Urbanización Montepríncipe, Boadilla del Monte, 28668 Madrid, Spain; 2 Departamento de Química, Universidad de La Rioja, C/Madre de Dios, 51, 26006 Logroño, Spain; 3 Departamento de Ciencias Farmacéuticas y de la Salud, Facultad de Farmacia, Universidad San Pablo-CEU, CEU Universities, Urbanización Montepríncipe, Boadilla del Monte, 28668 Madrid, Spain; 4 Departament de Ciència de Materials i Química Física and IQTC, Universitat de Barcelona, C/Martí i Franquès, 1, 08028 Barcelona, Spain

**Keywords:** hepatitis C virus, enzyme catalysis, concerted mechanism in serine protease reaction, NS3/NS4A serine protease, QM/MM reaction mechanism, EA-VTST/MT, rate constants, substrateNS4B/5A

## Abstract

Reaction mechanisms and rate constants of the acylation reaction of the hepatitis C virus (HCV) NS3/NS4A serine protease with the NS4B/5A natural substrate were studied using SCC-DFTB/MM (self-consistent charge density functional tight binding/molecular mechanics) and EA-VTST/MT (ensemble-averaged variational transition state theory/multidimensional tunneling) methods, considering the isotope effect (H/D). This reaction is crucial in the HCV life cycle. The reaction follows an essentially concerted mechanism. Although two elementary steps are involved, no intermediate step has been found between them. Thus, the proposed general two-step serine protease acylation mechanism, which includes a tetrahedral intermediate, does not occur here. This finding aligns with our studies on another natural substrate (NS5A/5B), indicating a greater variety in mechanism than previously expected. Tunneling and recrossing play an intermediate role; the activation free energy barriers are in good agreement with the experimental value, and the kinetic isotope effect (k(H)/k(D)) is somewhat larger than one (1.3). The rate constant value is not reproduced due to the exponential dependence of the rate constant on the activation free energy.

## 1. Introduction

Hepatitis C is a viral infection caused by the hepatitis C virus (HCV) that primarily affects the liver. As it is estimated that 71 million people are chronically infected with HCV worldwide [1], it remains a significant global health issue, mainly due to its potential to cause chronic liver diseases such as cirrhosis, hepatocellular carcinoma and the need for liver transplantation [2].

HCV is highly infectious and can be transmitted through contact with infected blood, including intravenous drug use, unsafe healthcare practices, and blood transfusions, until HCV screening implementation [3]. Screening for HCV typically involves detecting anti-HCV antibodies, followed by confirmatory HCV RNA, and early diagnosis is crucial for effective management and reducing transmission [4].

The introduction of Direct-Acting Antivirals (DAAs) has revolutionized HCV treatment, offering high sustained virologic response (SVR) rates exceeding 95% in most populations and offering cure rates above 90%, improving safety, tolerability, and treatment duration compared to older interferon-based therapies [5].

DAAs inhibit specific non-structural viral proteins necessary for HCV replication and are divided into 3 classes defined by their mechanism of action and target: NS3/4A protease inhibitors (drugs ending in -previr), non-nucleoside and nucleotide analog NS5B RNA-dependent RNA-polymerase inhibitors (drugs ending in -buvir), and NS5A inhibitors (drugs ending in -asvir) [6]. Current approved next-generation pan-genotypic regimens are Glecaprevir/Pibrentasvir (G/P), Sofosbuvir/Velpatasvir (SOF/VEL), and Sofosbuvir/Velpatasvir/Voxilaprevir (SOF/VEL/VOX), which are firmly established as the treatment standard for most genotypes and clinical circumstances [7]. Their widespread use has been documented in countries such as Spain, China, the United States, and throughout the European Union, supported by regulatory approvals and high sustained virologic response rates in both clinical trials and real-world settings [8,9].

Numerous studies have evaluated the efficacy, safety, and clinical outcomes of these DAAs. The effectiveness of G/P combination as an 8-week regimen for treatment-naïve, non-cirrhotic patients has been demonstrated, achieving SVR rates of 98–99% across all genotypes in clinical trials, but it is contraindicated in decompensated cirrhosis [10]. Co-formulated SOF/VEL is another pan-genotypic option effective in both cirrhotic and non-cirrhotic patients with high SVR, over 95% [11]. While pan-genotypic DAAs largely mitigate the impact of viral resistance in treatment-naïve patients, a small percentage of DAA-treated patients develop resistance-associated substitutions, leading to virological failure [12].

SOF/VEL/VOX combination is the first-line retreatment option for DAA-experienced patients, particularly those with resistance-associated substitutions, and patients with advanced liver disease or genotype 3 infection, achieving SVR rates as high as 96–99% [13].

Current treatment guidelines, such as those issued by AASLD-IDSA and EASL, recommend G/P and SOF/VEL for first-line use and SOF/VEL/VOX for retreatment [14].

Despite the effectiveness of DAAs, challenges persist. Genotype 3, historically the most difficult to treat, remains an area of active investigation. Until the HCV vaccine becomes a reality, barriers such as reinfections and resistance make it necessary to develop treatments based on other mechanisms of action [15].

The HCV genome encodes a polyprotein precursor that is cleaved into at least ten viral proteins that correspond to four structural proteins (core, E1, E2 and P7) and six non-structural proteins (NS2, NS3, NS4A, NS4B, NS5A and NS5B). The NS3 protein, with the NS4A cofactor, which rearranges the NS3 active site towards the optimal geometrical configuration for reaction [16], catalyzes the cleavage at the peptidic junctions NS3/4A, NS4A/4B, NS4B/5A, and NS5A/5B [17]. Therefore, targeting NS3/NS4A activity inhibition represents a principal approach in the development of novel therapeutics for HCV [18].

The NS3/NS4A active site comprises the His57-Asp81-Ser139 catalytic triad alongside residues 135–139, which contribute to the oxyanion-stabilizing group [19]. Nuclear magnetic resonance studies show that when an inhibitor is covalently connected to the NS3/NS4A structure [16], the oxyanion-stabilizing group creates hydrogen bonds between the hemiketal oxygen and the amide group of Ser-139 and Gly-137. This study also identifies Arg-155 as responsible for forming a shield for the solvent in order to protect the hydrogen bond between His-57 and Asp-81. Additionally, Arg-109 appears to play a role in substrate recognition and, along with Lys-136, has been utilized as a target in the development of inhibitors [20,21].

The NS3 protein contains a Zn^2+^ ion that is tetrahedrally coordinated by Cys-97, Cys-99, Cys-145, and a water molecule, which is also hydrogen bonded to His-149. Nevertheless, the role of this zinc is supposed to be structural and not catalytic because it is located quite far from the active site.

We have considered the reaction mechanism of the NS3/NS4A enzyme with its main substrates theoretically in several works [22,23,24,25,26]. Once the Michaelis complex is formed, the acylation process starts with the nucleophilic attack on the carbonylic carbon atom by the oxygen atom of the nucleophilic –OH group of Ser-139. The imidazole ring of His-57 is positioned so that it can deprotonate the –OH group. The Asp-81 side chain is oriented to stabilize the resulting positive charge on His-57 through a hydrogen bond. In other serine proteases [27,28], the formation of a tetrahedral intermediate occurs after the nucleophilic attack process. In previous studies using the AM1/MM theoretical method [22,23,24,25], this intermediate was detected and its geometry characterized. It has been proposed, as a general serine protease reaction mechanism, that this intermediate establishes the acylation reaction as a two-step process, with the nucleophilic attack as the rate-limiting one.

However, a recent theoretical study of the reaction mechanism between the NS3/NS4A enzyme and the NS5A/5B substrate, which increased the calculation level to SCC-DFTB/MM [26], showed that the intermediate does not exist and the acylation reaction becomes concerted, meaning the whole process must be considered to calculate the rate constant. Correcting the AM1/MM energy using MP2 energies and various basis sets [25] reduces both the stability of the tetrahedral intermediate and the acylation barrier height, making the reaction mechanism closer to a concerted one for the three main substrates.

Regardless of the theoretical approach [22,23,24,25,26], calculations indicate that nucleophilic attack breaks the peptide bond by overcoming a barrier, resulting in release of the N-terminus and formation of the acyl-enzyme. The catalyzed process concludes with the deacylation step, during which hydrolysis of the acyl-enzyme by a water molecule occurs. Following this, the substrate’s C-terminus is released, and the enzyme becomes available for another catalytic cycle.

The calculated barriers were compared with experimental values obtained from kinetic data for the cleavage of three synthetic substrates that resemble natural substrates, indicating that, whereas the AM1/MM method [22,23,24,25] overestimates the reaction barrier, the MP2-corrected [25] and the SCC-DFTB/MM results [26] are in better agreement with experiment, suggesting a near-concerted mechanism for these enzyme reactions.

Although other experimental measurements than those reported in ref. [29] have been performed using different substrates and inhibitors [30,31,32,33,34,35,36,37,38], the most interesting work for comparison purposes is the former one.

In this work, we have employed the SCC-DFTB/CHARMM22 QM/MM approach together with the Ensemble-Averaged Variational Transition State (EA-VTST) method, which also includes an estimation of multidimensional tunneling (MT) and recrossing effects, to calculate the rate constants of the reaction involving the NS3/NS4A protease and the NS4B/5A natural substrate, extending the kinetic study previously performed for the NS5A/5B substrate [26]. Moreover, a detailed study on the reaction mechanism is also performed to continue the description of the reaction mechanisms of the NS3/NS4A enzyme with its substrates.

## 2. Computational Methods

### 2.1. Initial Setup

Our research group previously generated initial NS3/NS4A-NS4B/5A structures, solvated with a 37 Å TIP3 water sphere, as used in earlier studies (see refs. [20,21,22]). These models, based on PDB 1DXP and adjusted for the enzyme–substrate complex, have demonstrated stability in MM and QM/MM simulations. The SCC-DFTB/CHARMM22 method [39] was applied to a 53-atom QM region, including the NS3 catalytic triad (His-57, Asp-81, Ser-139) and substrate atoms near the scissile bond, following previous protocols (see ref. [31] for review). The wave function of these atoms was calculated using the SCC-DFTB method [40,41], which offers high accuracy for biological molecules [42] and is about a thousand times faster than DFT methods [40] with medium-sized basis sets [43]. This efficiency makes it ideal for QM/MM studies that require extensive sampling of conformational space during reactions for free energy calculations. SCC-DFTB has been widely applied in QM/MM research involving biological systems (see reference [31] for details). The remaining 10,979 atoms were modeled with the CHARMM22 [44] force field for the MM portion of the system.

**Figure 1:**
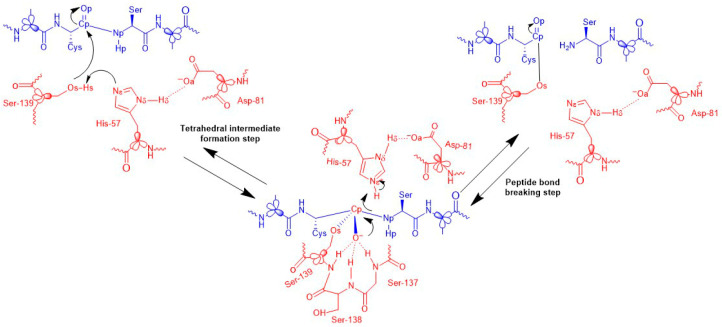
Proposed reaction mechanism for the acylation step catalyzed by the NS3 serine protease of HCV with the NS4B/NS5A substrate. The diagram illustrates the formation of the tetrahedral intermediate and subsequent peptide bond cleavage, highlighting the role of the catalytic triad residues—Ser139, His57, and Asp81. The figure includes a schematic representation of the quantum mechanical (QM) region used in the simulations, including the NS3 protease active site and the NS4B/5A substrate. Generalized hybrid orbital (GHO) frontier atoms are indicated as sp^3^ orbitals to denote the QM/MM boundary.

The Generalized Hybrid Orbital (GHO) method [45,46] was used for the five bonds at the QM-MM boundary, as shown in Figure 1. In this approach, electrostatic interactions were turned off, and van der Waals interactions were gradually diminished to zero between 12.0 and 13.5 Å. The MM region used a relative dielectric constant (ε) of one for electrostatics.

**Figure 2:**
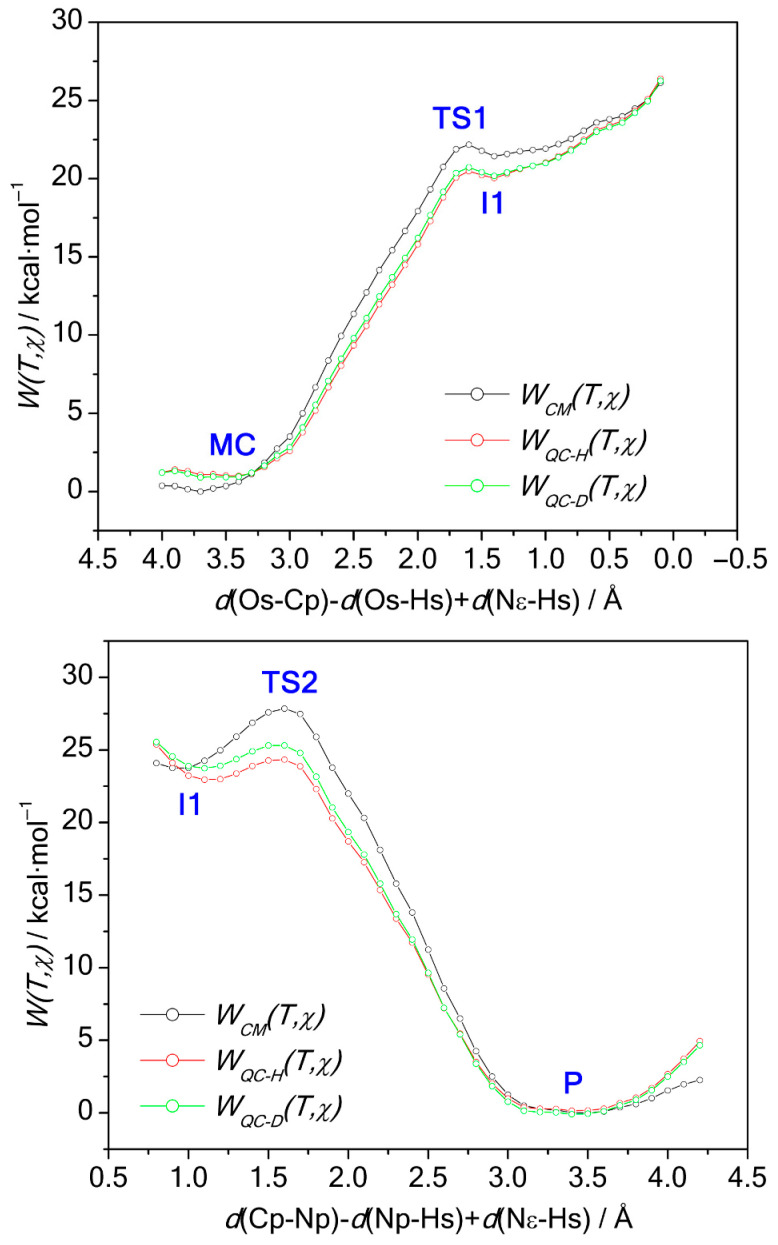
Classical (black) and quantum-corrected (quasiclassical) SCC-DFTB/MM free energy profiles are shown for both tetrahedral intermediate formation (**top**) and peptide bond cleavage (**bottom**) in the NS3/NS4A protease reaction with the NS4B/5A substrate. Key states—Michaelis complex (MC), transition states (TS1/TS2), intermediate (I1), and product (P)—are labeled, with all energies referenced to MC (set to zero). In the quantum-corrected profiles, proton transfer is highlighted in red and deuteron transfer in green.

The generally accepted serine protease reaction mechanism [28] involves two main steps: acylation, which forms an acyl-enzyme intermediate, and hydrolysis, which releases the enzyme and peptide fragments. We focus on the rate-limiting acylation process, consisting of a nucleophilic attack and peptide bond cleavage (see Figure 2), with key interatomic distances highlighted. Our results will compare the well-established two-step acylation mechanism [24]—characterized by a tetrahedral intermediate—with a concerted mechanism proposed for the NS3/NS4A + NS5A/5B reaction [26], where this intermediate may not form (see Figure 1).

### 2.2. QM/MM and Rate Constant Calculations

We follow the same protocol described in our previous NS3/NS4A + NS5A/5B theoretical study [26]. Below, we present a summary of the protocol followed, highlighting the parameters used in the present study. The initial Michaelis complex was taken from an equilibrated MM simulation. After defining QM and GHO atoms, SCC-DFTB/MM energy minimization with the Adapted Basis Newton–Raphson (ABNR) method [47] proceeded until the root mean square deviation of the gradient (GRMS) dropped below 1 × 10^−4^ kcal·mol^−1^·Å^−1^. Using this structure as a starting point, three reaction coordinates described the nucleophilic attack: (1) Os-Cp distance—oxygen of Ser-139 attacking substrate’s carbonyl carbon; (2) Os-Hs minus Hs-N distances—proton transfer; (3) antisymmetric combination of these coordinates (see Figure 1 for atom naming and spatial arrangement). The product characterization involved adjusting interatomic distances to model the acyl-enzyme intermediate, which became the starting structure for peptide bond cleavage simulations. Key reaction coordinates included (1) Cp-Np distance, (2) Np-Hs minus N-Hs differential (proton transfer), and (3) their antisymmetric combination offering a comprehensive perspective on both bond dissociation and proton transfer events.

System dynamics were simulated using the stochastic boundary molecular dynamics (SBMD) method [48] with a 26 Å buffer, 1 fs time step, SHAKE algorithm [49], and specified non-bonded interactions. Structures were heated for 100 ps to 300 K, equilibrated for 500 ps, and umbrella sampling was applied over discrete coordinates to calculate the potential of mean force (PMF) or classical free energy surface (FES). An umbrella potential (*V* = 50 kcal·mol^−1^·Å^−2^·Δ^2^) was used at 0.1 Å intervals, with each point undergoing 15 ps equilibration and 50 ps production (only production data contributed to PMF/FES). The Weighted Histogram Analysis Method [50,51] (WHAM) was then employed to remove the umbrella effect and extract thermodynamic profiles from the simulation results.

Using the Ensemble-Averaged Variational Transition State/Multidimensional Tunneling method [52,53,54], as in the previous NS3/NS4A + NS5A/5B study [26], we calculated rate constants for the processes examined. The variational transition state rate constant, *k^CVT^*(*T*), was determined from the quasiclassical free energy of activation, $\Delta G_{QC}^{‡}$, along with the recrossing transmission, *Γ*(*T*), and quantum tunneling, *κ*(*T*) coefficients, using the following formula:

(1) $$k^{CVT}\left(T\right)=\Gamma \left(T\right)\cdot \kappa (T)\cdot \frac{k_{B}\cdot T}{h}\cdot \exp\left(-\frac{\Delta G_{QC}^{‡}}{RT}\right)$$

where *k_B_*, *h* and *R* are Boltzmann’s, Planck’s, and ideal gas constants, respectively.

To facilitate comparison with experimental kinetic data, we use the phenomenological activation free energy (∆*G^‡^_phen_*(*T*)) that includes $\Delta G_{QC}^{‡}$ and free energy associated with recrossing transmission and quantum tunneling, which allows comparison with recrossing transmission ($\Delta G_{phen}^{exp}(T)$) determined by applying the classical Eyring equation to the experimentally measured rate constant (constant (*k^exp^*):

(2) $$k^{exp}=\frac{k_{B}\cdot T}{h}\exp\left[-\frac{\Delta G_{phen}^{exp}(T)}{R\cdot T}\right]$$

After determining the PMF, the classical activation free energy, *W_CM_*(*T*,*χ*), is refined for vibrational mode quantization (excluding the specified reaction coordinate). Representative samples from simulations are analyzed with generalized normal-mode analysis on selected QM atoms to derive the quasiclassical potential of mean force *W_QC_*(*T*,*χ*). The quasiclassical activation free energy, Δ*G^‡^_QC_*, is then calculated as:

(3) $$\Delta G_{QC}^{‡}(T)=W_{QC}(T,\chi^{‡})-\left[W_{QC}\left(T,\chi^{R}\right)+G_{F,QC}(T)\right]+\Delta C(\chi )$$

where *χ^‡^* represents the transition state and R the reactant state. Quantum mechanical vibrational free energy, *G_F_*_,*QC*_, and the Jacobian correction (usually omitted) are considered.

The analysis covered 6700 structures per substrate, using 100 structures per umbrella sampling window. The transmission coefficient, *γ*(*T*), was averaged over 25 reaction paths. Each path began with structures at the maximum Δ*G^‡^_QC_*(*T*), and minimum energy paths were computed for QM atoms within an MM potential [52,53]. These paths were used to estimate recrossing factors via the optimized multidimensional tunneling (OMT) approximation [55,56], which informed calculations of *k^CVT^*(*T*) and ∆*G^‡^_phen_*(*T*). The final energy term can be compared with experimental values as shown in Equation (2).

The kinetic isotope effect (KIE) for replacing H with D (see Figure S1) was estimated using a single PMF. We calculated *W_QC_*(*T*,*χ*) and determined recrossing and tunneling factors for both isotopes, yielding *k*(*H*)/*k*(*D*). Rate constants were computed with the CHARMMRATE program [57] using EA-VTST/MT theory.

## 3. Results and Discussion

### 3.1. SCC-DFTB/MM Classical Free Energy Summary

We initially explored the potential energy surface (PES) and calculated minimum energy paths to prepare for free energy calculations. Figures S3–S5 and Table S2 in the Supporting Information detail the structures and energies of stationary points, highlighting changes in key interatomic distances among intermediates. While these materials provide valuable context, our focus is on the free energy results discussed in the main text. Using the setup described earlier, we varied combinations of interatomic distances and calculated the classical PMF at 300 K for nucleophilic attack and peptide bond breaking, as shown in Figure 2. Table 1 presents the stationary point energies from WHAM analysis, along with data from a previous study for comparison.

**Figure 3:**
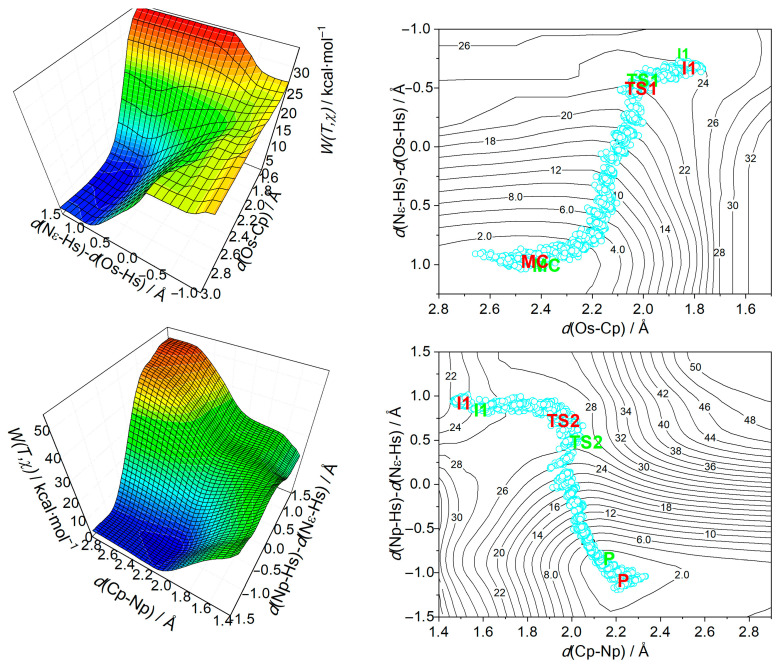
SCC-DFTB/MM free energy maps illustrate the catalytic reaction of hepatitis C virus NS3/NS4A protease with its substrate. The top panel shows tetrahedral intermediate formation, while the bottom shows peptide bond cleavage. Both panels have 3D and 2D plots marking reactant (R), transition states (TS1, TS2), Michaelis complex (MC), and product (P); red and green highlight actual and published reaction paths. Cyan circles are representative structures along the minimum energy path.

Figure 2 shows a barrier, TS1, at 21.3 kcal·mol^−1^ above the Michaelis complex. Just below TS1 is intermediate I1, with a free energy 0.1 kcal·mol^−1^ lower. After I1, another barrier, TS2, appears at 24.5 kcal·mol^−1^ before acyl-enzyme formation, making the energy gap between I1 and TS2 3.3 kcal·mol^−1^. Unlike potential energy data (see Supplementary Materials), no barrier exists for I1 formation, so TS2 marks the main barrier in acylation (see Figure S4 for details). The reaction mechanism is further clarified by three-dimensional free energy surfaces in Figure 3, which include umbrella sampling simulation structures on equi-free energy contour plots (right panel), providing insight into system evolution during the reaction.

Comparing the energy values of the two substrates for the stationary points obtained, we see that for the first stage of the process, these are energetically higher. The first barrier is 0.7 kcal/mol higher, while we find an intermediate that could be considered labile since its difference is 0.1 kcal/mol, as in the previous case. However, when considering the peptide bond formation process, we find a lower barrier (3.3 kcal/mol from the intermediate for the NS4B/5A substrate, while it is 4.7 kcal/mol for the NS5A/5B substrate). Furthermore, we see that for this substrate, the energy of the products is negative, which suggests that we are dealing with an exothermic process, which is generally more favored from a thermodynamic point of view.

During the nucleophilic attack, proton transfer occurs in a concerted manner as Ser-139’s oxygen approaches the substrate’s carbonyl carbon. The peptide bond breaks before reaching the TS2 barrier, after which a second proton transfer takes place via a near two-step mechanism—consistent with prior findings for the NS5A/5B substrate. The stationary point positions of NS5A/NS5B and NS4B/NS5A substrates were compared to demonstrate structural changes. Both share similar reaction coordinates during tetrahedral intermediate formation but differ significantly during peptide bond cleavage due to variations in reaction energetics and stabilization of the products.

### 3.2. Quasiclassical Free Energy Profiles and Reaction Mechanism

While quantum methods calculate the energy and forces for the QM region, nuclear motion is treated classically via Newton’s equations. To account for this, a quantum correction is applied to the PMF by evaluating the vibrational normal modes of selected structures along the PMF (see Equation (5) in refs. [24,58]). This enables assessment of H/D substitution in Ser-139 for KIE calculation. Details are provided in Table S1 and Figure S2 of the SI. The resulting quasiclassical free energy profiles (W_QC_), reflecting quantum corrections and both isotope substitutions, appear in Figure 2.

Quantum correction generally reduces free energy across all processes studied. For the NS4B/5A substrate—a cleavage site processed by HCV NS3/NS4A protease—the barrier to forming the tetrahedral intermediate is almost eliminated, though a shallow I1 intermediate remains. This substrate displays distinctive reactivity due to its sequence, notably a proline at P2, which affects catalysis [24]. NS4B/5A cleavage also relies on the NS4A cofactor for stabilizing the protease–substrate complex and enhancing activity [59,60]. Energy minimization of I1 structures consistently yields Michaelis complex geometries, confirming I1’s instability. These results are consistent with earlier QM/MM studies emphasizing I1’s dynamic nature and dependence on substrate interactions [24,61].

In the initial nucleophilic attack (or tetrahedral intermediate formation), there is little difference in corrected free energy profiles between H+ and D+ transfers, indicating minimal isotopic effect due to a concerted atom transfer, consistent with the Os-Cp approach (see Figure 3, top panels). While some studies report a concerted mechanism for NS3/NS4A [26] and trypsin reactions [38,39], others propose a stepwise process for chymotrypsin [62,63]. During peptide bond cleavage (Figure 3, lower panels), the C-N bond breaks en route from the intermediate to TS2, with significant isotopic substitution occurring as TS2 converts to the acyl-enzyme product—similar to the NS5A/5B substrate case [26]. Proton transfer displays a lower corrected energy profile, leading to faster reactivity. Typically, serine protease mechanisms involve a tetrahedral intermediate after nucleophilic attack. Figure 2 shows this intermediate in the free energy profile, even post-quantum correction, suggesting a somewhat stepwise process. However, the intermediate remains highly unstable, as minimization efforts consistently revert to reactant geometries.

The experimental evidence of intermediate formation is limited. Only stable structures have been observed under very special experimental conditions [64,65,66]. Several available drugs against HCV act as serine traps of the NS3/NS4A protease [67,68,69,70]. All evidence indicates that the tetrahedral intermediate has a very short lifetime, which is related to the efficiency of the substrate. The lifetime decreases with enhanced leaving group ability, and the reaction proceeds via a concerted mechanism when the intermediate’s lifetime becomes shorter than a vibrational period [28].

For the NS4B/5A substrate, an extremely short-lived intermediate may occur during nucleophilic attack, but it cannot be characterized. This suggests a concerted reaction mechanism bordering on stepwise. NS4B/5A is highly efficient, with a lifetime just under a vibrational period (~10^−13^ s). The acylation mechanisms for NS4B/5A and NS5A/5B are very similar. Both start when Ser-139’s oxygen atom attacks the carbonyl carbon of the scissile bond, followed by a proton transfer to His-57’s ε nitrogen. This creates a positive charge in the histidine ring, which is stabilized by a hydrogen bond with Asp-81. These steps happen simultaneously. As the peptide bond cleaves, the process reaches the acylation transition state without any stable intermediates. The nitrogen from the bond bonds with a proton from His-57, resulting in substrate amino termination and formation of acyl-enzyme.

### 3.3. Rate Constant Calculations and Kinetic Analysis

To determine the rate constant, transmission coefficients for tunneling (κ) and recrossing (Γ) were averaged over 25 values per isotope at 300 K. These coefficients were calculated using minimum energy paths from transition state geometries to the Michaelis complex and acyl-enzyme species. Only the TS2 ensemble yielded successful calculations; TS1 structures were not recognized as transition states. Thus, the same point was used as the reaction barrier in Equation (1), highlighting a concerted reaction mechanism.

Table 2 presents quasiclassical free energy, phenomenological free energy (for comparison with experimental data), calculated and experimental rate constants, and kinetic isotope effect (KIE) factors.

For the studied substrate, the tunneling transmission coefficient is higher for proton transfer than for deuteron transfer. The rate constants increase by about 100% for H^+^ and 60% for D^+^, resulting in decreases of 0.4 kcal·mol^−1^ for H^+^ and 0.3 kcal·mol^−1^ for D^+^. These reductions indicate that tunneling lowers the energy needed for both transfers.

Recrossing factors indicate the probability of a molecule crossing a transition state multiple times, with values of 0.56 for light isotope transfer and 0.64 for heavy. At 300 K, the free energy barrier increases by 0.3 kcal·mol^−1^ (H^+^/D^+^). Like the NS5A/5B substrate [26], these results imply the reaction is concerted, meaning all steps happen simultaneously.

When tunneling and recrossing are considered together, overall transmission coefficients are 1.13 for H^+^ and 1.02 for D^+^. This lowers the free energy barrier by 0.1 kcal·mol^−1^ for H^+^, with no effect for D^+^. Here, unlike with the NS5A/5B substrate, the classical Eyring equation sufficiently describes H^+^ and D^+^ transfers, as tunneling and recrossing effects do not counterbalance each other [26].

High standard deviation in kinetic parameters reflects the diversity of transition states, each with distinct energy barriers and configurations. These variations are influenced by fluctuating temperature, pressure, and molecular interactions at the reactive center. The calculated phenomenological activation free energies, ∆*G^‡^_phen_*(*T*), are 20.2 kcal·mol^−1^ for H^+^ transfer and 20.4 kcal·mol^−1^ for D^+^, indicating the respective energy barriers. Lower values suggest a more favorable and likely transfer process.

Based on the tunneling and recrossing factors along with Equation (1), the calculated CVT rate constant for the H^+^ transfer process at 300 K is *k^CVT^*(H^+^, 300 K) = 0.77 min^−1^, while the corresponding value for D^+^ transfer is *k^CVT^*(D^+^, 300 K) = 0.48 min^−1^. These rate constants clarify reaction kinetics—higher values mean faster reactions. The resulting KIE of 1.60 (just above 1.0) indicates that H^+^ transfer proceeds more readily than D^+^, reflecting a lower activation free energy for H^+^. Note that the free energy difference is the main reason for obtaining a KIE value greater than one, because the <κ>·<Γ> term is quite similar for both isotopes.

The free energy value for the H^+^ isotope variant (20.2 kcal·mol^−1^) is close to the experimental activation free energy (18.6 kcal·mol^−1^) [29], with the theoretical result only 1.6 kcal·mol^−1^ (8.6%) higher, within expected QM/MM calculation [68,69] accuracy. Despite this small free energy difference, the exponential relationship with rate constants leads to significant variation: the calculated k^CVT^(300 K) is 0.77 min^−1^, while the experimental k^exp^(296 K) is 11.00 min^−1^, about an order of magnitude larger.

In Table 3, the experimental [29] and calculated rate constants for the reaction of NS3/NS4A with NS5A/5B [26] and NS4B/5A substrates are provided for a general comparison between the experiments and the EA-VTST/MT results based on the SCC-DFTB/MM calculations.

The table shows that both experimental and calculated results indicate a higher reaction velocity with the NS4B/5A substrate than with NS5A/5B. However, significant differences exist between the experimental and theoretical rate constants [29], revealing limitations in the simulation models and the need for refinement to better represent natural substrates. The natural sequences are EDVVCCSMSY (substrate 1) and ECTTPCSGSW (substrate 2), while the tested sequences include additional residues: EDVVCCSMSY**K** and **WISS**ECTTPCSGSW**LRDIWD** (added residues in bold).

Agreement improves when comparing phenomenological free energy barriers: for substrates 1 and 2, the observed differences are 2.3 and 1.6 kcal·mol^−1^, which fall within the typically accepted 1 kcal·mol^−1^ accuracy range for comparing experimental and calculated enzyme reaction barriers. These correspond to relative errors of 12.2% and 8.6%.

Our simulations suggest that resistance-associated amino acid substitutions (RASs), especially in NS3 and NS5A, may affect energetic and structural behavior. Mutations like Y93H and L31M/V in NS5A [70,71] and R155K and D168V in NS3 [72] can alter replication, decrease drug sensitivity, and influence protein dynamics. Although we did not model these mutations directly, their effects on enzyme function and drug response highlight the need to consider mutational variation when analyzing mechanistic and energetic profiles.

## 4. Conclusions

We investigated the acylation reaction mechanism and rate constant of HCV NS3/NS4A serine protease with its natural substrate NS4B/5A, vital to the hepatitis C virus life cycle. Using a QM/MM (SCC-DFTB/CHARMM22) approach, we mapped the quantum-corrected free energy surface between reactants and products and calculated the rate constant via transition state theory (EA-VTST/MT), also considering isotope effects (H/D). Our analysis reveals a concerted reaction mechanism, with no intermediate found between nucleophilic attack and peptide bond breaking, challenging the commonly accepted two-step serine protease acylation involving a tetrahedral intermediate. Similar results were observed for another substrate, NS5A/5B, underscoring greater mechanistic diversity in serine proteases than previously thought.

Although experimental and theoretical rate constants differ without statistical evaluation, the calculated activation free energy barrier (20.2 kcal mol^−1^) is close to the experimental value (18.6 kcal mol^−1^), supporting our model’s accuracy. The observed reactivity order (NS4B/5A > NS5A/5B) matches experiments, but the rate constant’s exponential dependence on activation energy limits its reliability. Tunneling doubles reactivity, while recrossing reduces it by 40%. The KIE (k(H)/k(D)) is 1.6, compared to 2.0 for NS5A/5B. Further details on these interactions would improve clarity.

The reaction mechanism for serine proteases, including NS3/NS4A, has been studied. Findings indicate that the acylation step in the NS3/NS4A + NS5A/5B system can be characterized as a pre-association uncoupled concerted reaction. This suggests that, while the process involves both nucleophilic attack and bond rupture, no barrier for intermediate formation or evidence of a stable intermediate was observed.

This work continues our SCC-DFTB/MM theoretical analysis, which combines quantum mechanics and molecular mechanics, of the reaction mechanism and kinetics of the main reactions of the HCV NS3/NS4 serine protease, which is one of the main targets in the fight against the hepatitis C virus infection. In addition to its theoretical interest, our characterization of the transition state structure TS2 can aid in designing novel NS3/NS4A inhibitors. Our findings provide valuable insights into the reaction mechanism and kinetics of the HCV NS3/NS4A serine protease. This knowledge can significantly contribute to the development of new NS3/NS4A inhibitors, which are crucial for creating effective treatments for hepatitis C virus infection.

## Figures and Tables

**Table 1:** The free energy of the stationary points referred to the Michaelis Complex (MC), at the classical level, without quantum corrections for the actual study and the previous work [26].

	ΔG_CM_/kcal·mol^−1^	
	NS4B/5A	NS5A/5B [26]
MC	0.0	0.0
TS1	21.3	20.6
I1	21.2	20.5
TS2	24.5	25.2
P	−2.3	1.8

**Table 2:** Kinetic parameters of NS3/NS4A-mediated acylation with NS4B/5A.

	Proton	Deuteron	Exp. [29]
<κ>	2.02 ± 1.23	1.59 ± 0.84	-
<Γ>	0.56 ± 0.39	0.64 ± 0.37	-
<κ>·<Γ>	1.13 ± 1.05	1.02 ± 0.79	-
Δ*G^‡^_QC_*/kcal·mol^−1^	20.2	20.4	
Δ*G^‡^_phen_*/kcal·mol^−1^	20.2	20.4	18.6 *
k^CVT^/min^−1^	0.77	0.48	11.00
k^CVT^(*H*^+^)/k^CVT^(*D*^+^)	1.60		

* The value is derived from an experimental kcat of 11.00 min^−1^ for H^+^ (T = 296 K), obtained using a synthetic substrate analogous to the NS4B/5A substrate. The estimated experimental error ranges from 10 to 30%. Theoretical data are provided at 300 K.

**Table 3:** Experimental and calculated rate constants and phenomenological free energies for the reaction of the NS3/NS4 protease with its main substrates.

Substrate	*k^CVT^*(300 K)/min^−1^	*k^exp^*(296 K)/min^−1^	Δ*G^‡^_phen_*/kcal·mol^−1^	
			Theoretical	Exp. [29]
NS5A/5B [26]	0.20	6.26	21.0	18.7
NS4B/5A	0.77	11.00	20.2	18.6

## Data Availability

The original contributions presented in this study are included in the article/Supplementary Materials. Further inquiries can be directed to the corresponding author.

## Supplementary Materials

The following supporting information can be downloaded at: https://www.mdpi.com/article/10.3390/biom15111619/s1, Figure S1. Fragments of the substrates (up) and residues of the NS3/NS4A protease (down) included in the QM region; Figure S2. ΔWvib values obtained and polynomial fit for the nucleophilic attack (top) and peptide bond breakage (down) steps of the reaction of the NS3/NS4A protease with the NS4B/5A substrate, considering the proton (right) and deuteron (left) transfer; Figure S3. SCC-DFTB/MM potential energy profiles the nucleophilic attack (up) and peptide bond breakage (down) for the reaction of the NS3/NS4A protease with the NS4B/5A substrate; Figure S4. SCC-DFTB/MM potential energy surface regions for the nucleophilic attack (up) and peptide bond breakage (down) for the reaction of the NS3/NS4A protease with the NS4B/5A substrate; Figure S5. SCC-DFTB/MM structures of the QM region for the Michaelis complex (MC), intermediates (I1a and I1b), and products (P) of the NS3/NS4A + NS4B/5A reaction; Figure S6. Overlapping of the QM region for the structures of the intermediates; Table S1. Fitting polynomial coefficients of the vibrational correction for the nucleophilic attack and peptide bond breaking steps of the NS3/NS4A protease with the NS4B/5A substrate; Table S2. SCC-DFTB/MM potential energies of the stationary points of the NS3/NS4A + NS4B/5A reaction, referred to the Michaelis complex (MC).
