# Supplementary material for: Mechanistic and Kinetic Insights into the Acylation Reaction of Hepatitis C Virus NS3/NS4A Serine Protease with NS4B/5A Substrate
Source: Biomolecules. 2025 Nov 18;15(11):1619. doi: 10.3390/biom15111619 (PMC12650507; doi:10.3390/biom15111619)
Supplement: Supplementary file 1 [file biomolecules-15-01619-s001.zip › biomolecules-3787571-supplementary.pdf]

Supporting Information

Mechanistic and Kinetic Insights into the Acylation Reaction of Hepatitis C Virus NS3/NS4A Serine Protease with NS4B/5A Substrate.

José Ángel Martínez-González,[a,b,*] Nuria Salazar[c], Rodrigo Martínez.[b], Miguel González,[d] &

[a] Departamento de Química y Bioquímica, Facultad de Farmacia, Universidad San Pablo-CEU, CEU Universities, Urbanización Montepríncipe, 28668 Boadilla del Monte, Madrid, (Spain).

[b] Departamento de Química, Universidad de La Rioja, C/ Madre de Dios, 51. 26006 Logroño (Spain).

c Departamento de Ciencias Farmacéuticas y de la Salud Facultad de Farmacia, Universidad San Pablo-CEU, CEU Universities, Urbanización Montepríncipe, 28668 Boadilla del Monte, Madrid, Spain

[d] Departament de Química Física i IQTC, Universitat de Barcelona, C/ Martí i Franquès, 1. 08028 Barcelona (Spain).

[*] jose.martinezgonzale1@ceu.es

S1. Vibrational data regarding the H/D Ser-139 isotopic substitution

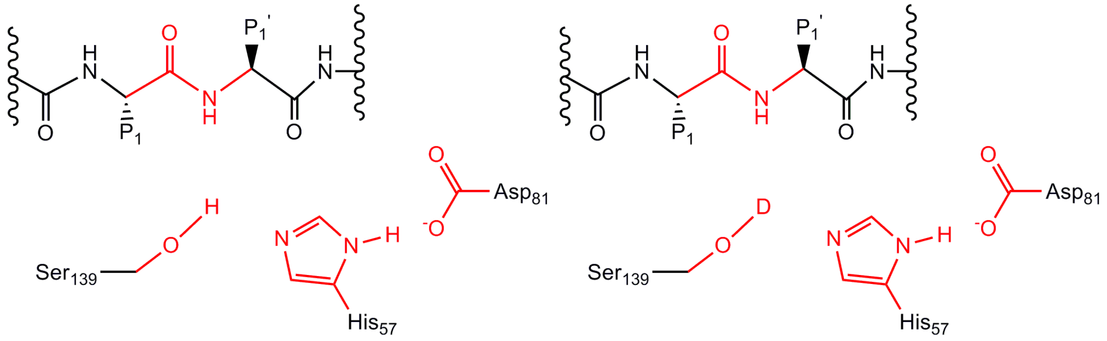

Figure S1. Fragments of the substrates (up) and residues of the NS3/NS4A protease (down) included in the QM region. The atoms in red are considered in the calculations of the vibrational frequencies and the H (left)/D(right) isotopic substitutions can be seen in the Ser-139 residue.

Table S1. Fitting polynomial coefficients of the vibrational correction for the nucleophilic attack and peptide bond breaking steps of the NS3/NS4A protease with the NS4B/5A substrate.

$$\Delta\omega_{vib} = a_0 + a_1 \cdot \chi + a_2 \cdot \chi^2 + a_3 \cdot \chi^3 + a_4 \cdot \chi^4 + a_5 \cdot \chi^5$$

Coefficient		a_0	a_1	a_2	a_3	a_4	a_5
Acylation	proton	-0.5365	-3.3579	-4.6610	-3.9174	1.3516	-0.1525
	deuteron	-0.3870	-3.1105	3.8957	-2.9646	0.9849	-0.1092
Bond break	proton	6.6201	2.7523	-22.1435	16.1303	-4.2744	0.3903
	deuteron	2.3625	11.0676	-25.6787	16.0018	-3.9818	-0.3520

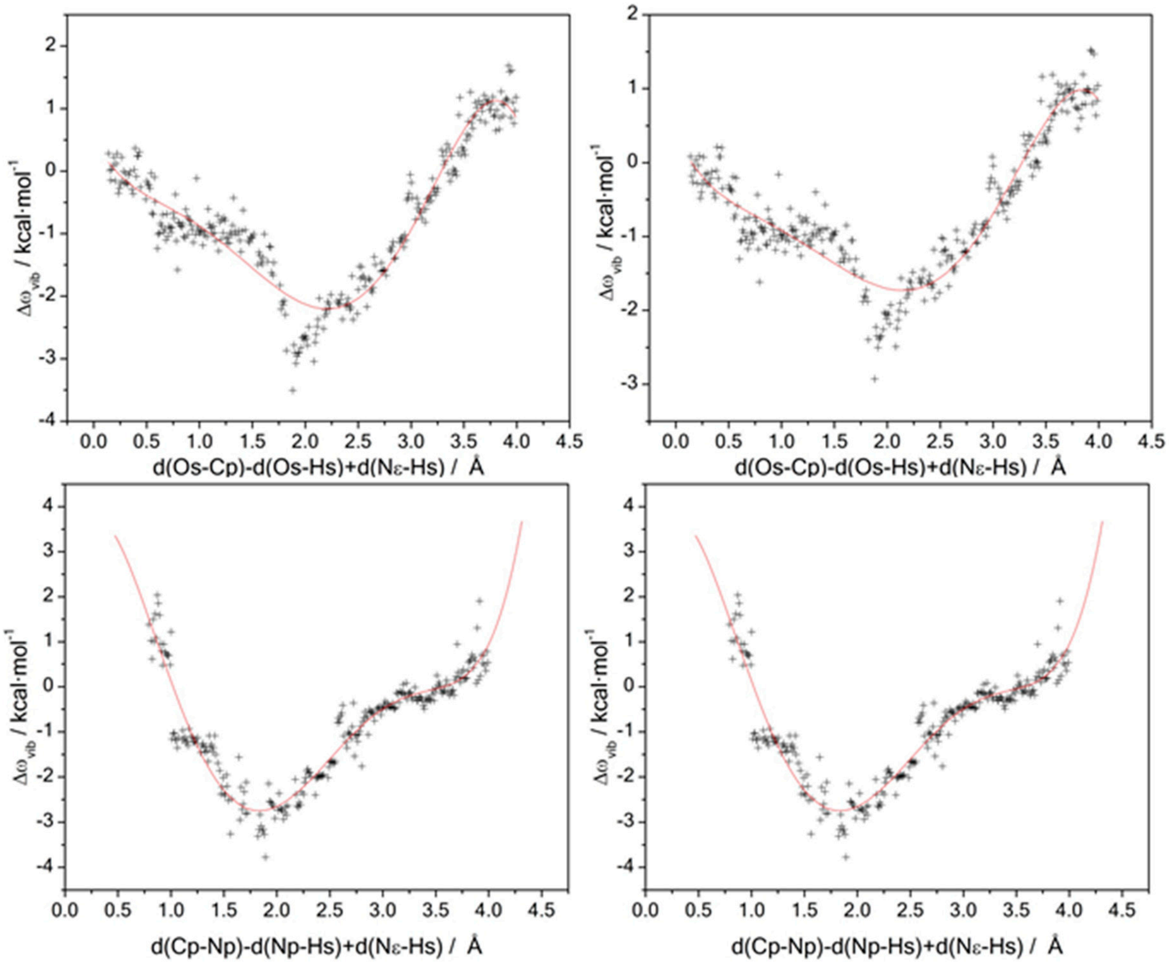

Figure S2. ΔW_{vib} values obtained and polynomial fit for the nucleophilic attack (top) and peptide bond breakage (down) steps of the reaction of the NS3/NS4A protease with the NS4B/5A substrate, considering the proton (right) and deuteron (left) transfer.

S2. SCC-DFTB/MM potential energy results

The SCC-DFTB/MM potential energy profile (minimum energy path) of the nucleophilic attack was calculated by varying the d(Os-Cp)-d(Os-Hs)+d(Nε-Hs) combination of distances from 3.9 Å to 0.0 Å. The corresponding profile for the peptide bond breakage was determined by varying the d(Cp-Np)-d(Np-Hs)+d(Nε-Hs) combination of distances from 0.70 Å to 4.50 Å. The results are presented in Figure S3.

The potential energy surface was calculated at the same level using the *d*(Os-Cp) and *d*(Nε-Hs)-*d*(Os-Hs) coordinates to describe the nucleophilic attack step, and the *d*(Cp-Np) and d(Np-Hs)-*d*(Nε-Hs) coordinates to describe the peptide bond breakage step (Figure S4)

Neither potential energy barriers nor minima (other than the Michaelis complex) are found in the figures corresponding to the nucleophilic attack step. The I1a point in the figures is marked where the slope of the potential energy changes. In the peptide bond breakage step an additional slope change is observed (I1b) that is followed by a potential energy barrier (TS2) leading to the acylation products.

All unrestrained minimization attempts carried out in order to characterize the I1a and I1b structures failed, as they led to the Michaelis complex. Hence, there is not a stable (tetrahedral) intermediate in the acylation process on the SCC-DFTB/MM potential energy surface.

The SCC-DFTB/MM potential energies, relative to the Michaelis complex, for I1a, I1b, TS2 and the products are given in Table S2; whereas the structures of the QM region for all stationary points (with the main interatomic distances shown) are presented in Figure S5. Furthermore, it can be seen that the structures of the intermediates I1a and I1b are very similar (see Figure S6). The difference between them is an umbrella motion of the Np atom which in the I1b structure is better oriented to get the Hs specie.

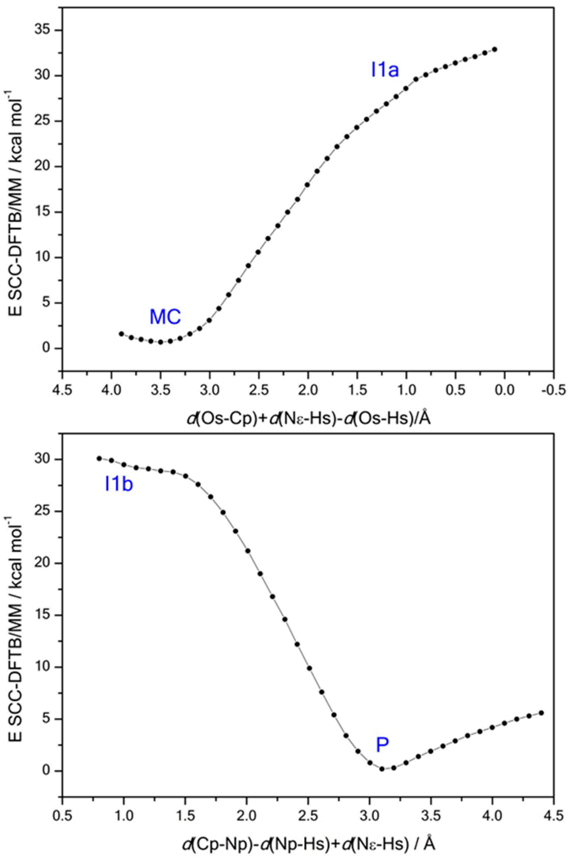

Figure S3. SCC-DFTB/MM potential energy profiles the nucleophilic attack (up) and peptide bond breakage (down) for the reaction of the the NS3/NS4A protease with the NS4B/5A substrate. The zero of energy is taken at the Michaelis complex (MC).

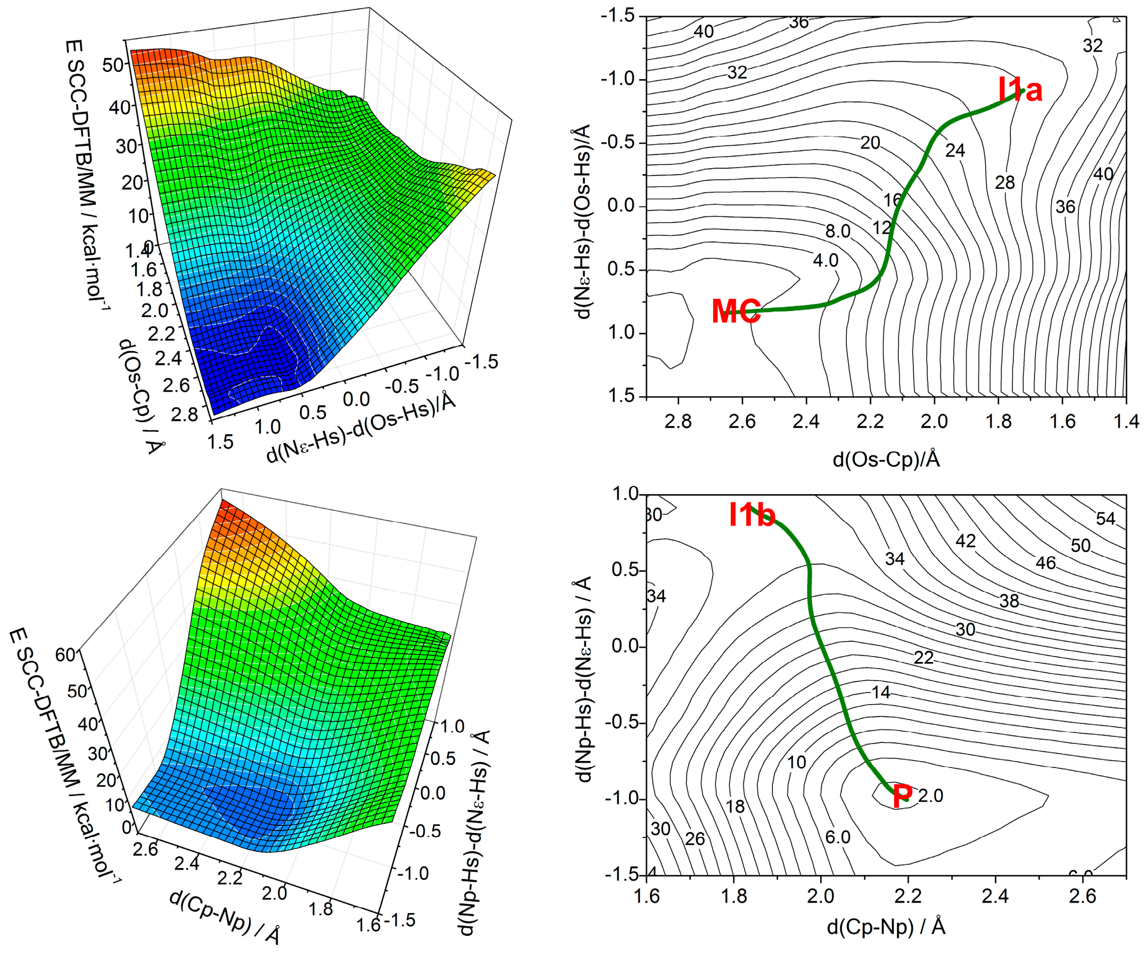

Figure S4. SCC-DFTB/MM potential energy surface regions for the nucleophilic attack (up) and peptide bond breakage (down) for the reaction of the NS3/NS4A protease with the NS4B/5A substrate. The minimum energy path of the two PES regions is represented by the green line in the equipotential contour plots representation (right).

Table S2. SCC-DFTB/MM potential energies of the stationary points of the NS3/NS4A + NS4B/5A reaction, referred to the Michaelis complex (MC).

	ΔE/kcal·mol^{-1}
MC	0.00
TS1	-
I1a	28.60
I1b	29.10
TS2	-
P	0.20

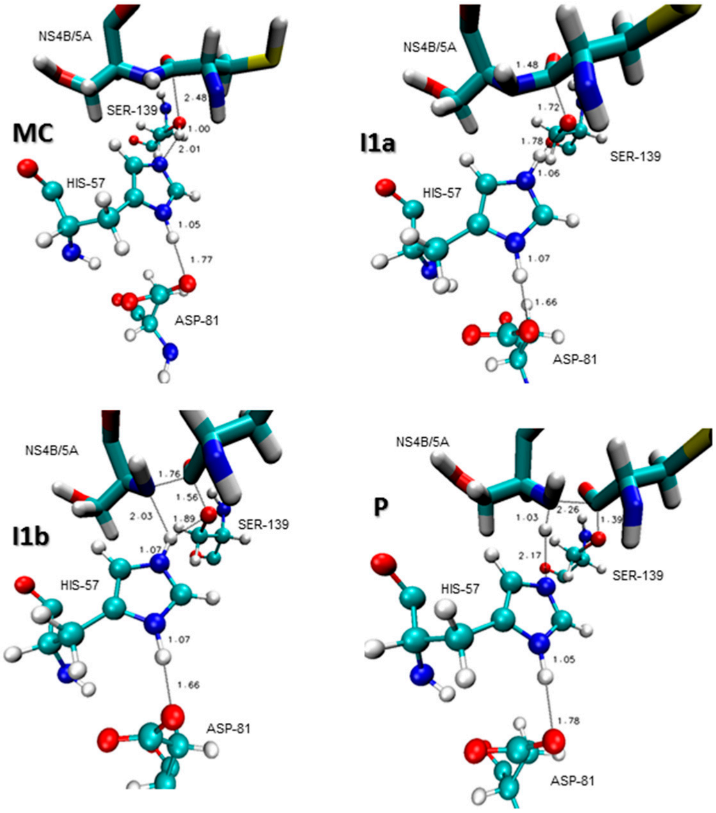

Figure S5. SCC-DFTB/MM structures of the QM region for the Michaelis complex (MC), intermediates (I1a and I1b), and products (P) of the NS3/NS4A + NS4B/5A reaction.

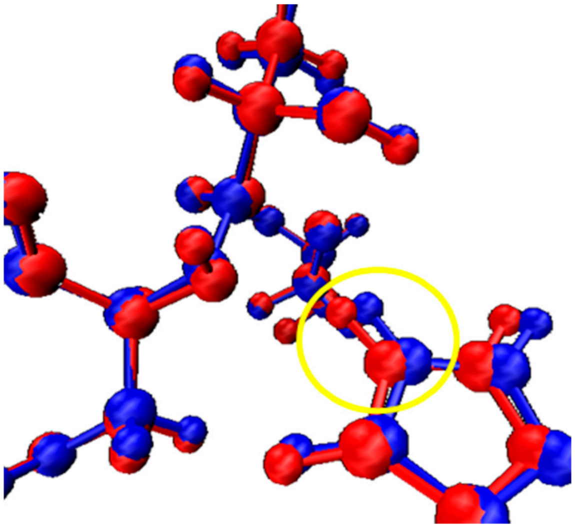

Figure S6. Overlapping of the QM region for the structures of the intermediates I1a (blue) and I1b (red) for the reaction of the NS3/NS4A protease with the NS4B/5A substrate, characterized at the SCC-DFTB/MM level. The His-57 residue movement has been highlighted (yellow circle).
